# Publicly Available Dental Image Datasets for Artificial Intelligence

**DOI:** 10.1177/00220345241272052

**Published:** 2024-10-18

**Authors:** S.E. Uribe, J. Issa, F. Sohrabniya, A. Denny, N.N. Kim, A.F. Dayo, A. Chaurasia, A. Sofi-Mahmudi, M. Büttner, F. Schwendicke

**Affiliations:** 1 Department of Conservative Dentistry and Oral Health, Riga Stradins University, Riga, Latvia; 2 Baltic Biomaterials Centre of Excellence, Headquarters at Riga Technical University, Riga, Latvia; 3 Chair of Practical Clinical Dentistry, Department of Diagnostics, Poznań University of Medical Sciences, Poznań, Poland; 4 Doctoral School, Poznań University of Medical Sciences, Poznań, Poland; 5 Topic Group Dental Diagnostic and Digital Dentistry, ITU/WHO Focus Group AI on Health, Berlin, Germany; 6 Independent researcher, Ramstein, Germany; 7 College of Arts and Sciences, University of Pennsylvania, Philadelphia, PA, USA; 8 Department of Oral Medicine, University of Pennsylvania School of Dental Medicine, Philadelphia, PA, USA; 9 Department of Oral Medicine & Radiology, King George’s Medical University, Lucknow, Uttar Pradesh, India; 10 Department of Health Research Methods, Evidence, and Impact, McMaster University, Hamilton, ON, Canada; 11 National Pain Centre, Department of Anesthesia, McMaster University, Hamilton, ON, Canada; 12 Charité Universitätsmedizin, Berlin, Germany; 13 Clinic for Conservative Dentistry and Periodontology, LMU University Hospital, LMU Munich, Germany

**Keywords:** panoramic radiography, metadata, diagnostic imaging, artificial intelligence, big data, deep learning/machine learning, digital imaging/radiology

## Abstract

The development of artificial intelligence (AI) in dentistry requires large and well-annotated datasets. However, the availability of public dental imaging datasets remains unclear. This study aimed to provide a comprehensive overview of all publicly available dental imaging datasets to address this gap and support AI development. This observational study searched all publicly available dataset resources (academic databases, preprints, and AI challenges), focusing on datasets/articles from 2020 to 2023, with PubMed searches extending back to 2011. We comprehensively searched for dental AI datasets containing images (intraoral photos, scans, radiographs, etc.) using relevant keywords. We included datasets of >50 images obtained from publicly available sources. We extracted dataset characteristics, patient demographics, country of origin, dataset size, ethical clearance, image details, FAIRness metrics, and metadata completeness. We screened 131,028 records and extracted 16 unique dental imaging datasets. The datasets were obtained from Kaggle (18.8%), GitHub, Google, Mendeley, PubMed, Zenodo (each 12.5%), Grand-Challenge, OSF, and arXiv (each 6.25%). The primary focus was tooth segmentation (62.5%) and labeling (56.2%). Panoramic radiography was the most common imaging modality (58.8%). Of the 13 countries, China contributed the most images (2,413). Of the datasets, 75% contained annotations, whereas the methods used to establish labels were often unclear and inconsistent. Only 31.2% of the datasets reported ethical approval, and 56.25% did not specify a license. Most data were obtained from dental clinics (50%). Intraoral radiographs had the highest findability score in the FAIR assessment, whereas cone-beam computed tomography datasets scored the lowest in all categories. These findings revealed a scarcity of publicly available imaging dental data and inconsistent metadata reporting. To promote the development of robust, equitable, and generalizable AI tools for dental diagnostics, treatment, and research, efforts are needed to address data scarcity, increase diversity, mandate metadata completeness, and ensure FAIRness in AI dental imaging research.

## Introduction

Artificial intelligence (AI) has transformative potential for advancements in dental diagnostics and treatment (Schwendicke et al. 2020). The development, implementation, and validation of AI depends on the availability of large, well-annotated datasets (Ma et al. 2022). Dataset size, quality, and diversity are critical determinants of AI model training, validation, and benchmarking (Mongan and Halabi 2023), while creating and curating datasets pose significant challenges related to expertise, patient data management, and security.

Although the availability of publicly accessible medical imaging datasets in other fields, such as ophthalmology (Khan et al. 2021), is rapidly expanding, with thousands to millions of images (Mongan and Halabi 2023) being available, the state of dataset availability in dentistry remains unknown and underexplored. The exploration of AI datasets in medicine has shown significant disparities in geographic representation (Celi et al. 2022) and has raised concerns about algorithmic bias perpetuating existing health disparities due to inequalities in dataset creation and curation (Arora et al. 2023) and the limited generalizability of medical AI. In dentistry, existing meta-research has focused on developing AI applications with high performance on selected datasets, while only limited attention has been paid to exploring the data used to train and test AI models (Sengupta et al. 2022).

However, this research was limited to academic databases, which may not fully capture AI-specific data requirements such as ability, accessibility, interoperability, and reusability (FAIR) principles (Wilkinson et al. 2016). Because less than 2% of dental studies share datasets in a machine-actionable format (Uribe et al. 2022), exploring alternative sources to overcome this limitation and ensuring the availability and quality of AI-ready datasets is critical. Furthermore, AI deployment in dentistry is hindered by restricted access to large-scale clinical imaging data often situated within institutions (Schwendicke and Krois 2022). Assessing dental imaging dataset characteristics is vital for understanding the AI model training, validation, and benchmarking utility of AI models. Although radiology has been a significant AI development focus owing to the extensive imaging datasets, comprehensive information on the availability and characteristics of dental datasets is lacking.

The primary aim of this study is to identify and describe dental imaging datasets for AI model development and training and to evaluate their quality from a machine perspective. In addition, we will create a centralized directory containing dataset sources, accessibility, and summaries of populations, diseases, and imaging types to facilitate the understanding and use of dental imaging data in AI research and promote reliable advancements in dentistry for improved clinical outcomes and research.

## Methods

### Study Design and Registration

This observational study adhered to the STROBE guidelines. It was registered in the OSF (September 6, 2022; DOI 10.17605/OSF.IO/HUZ72) and conducted as an activity of the WHO/ITU/WIPO Global Initiative AI for Health (World Health Organization 2023).

### Settings

This study aimed to characterize publicly available datasets from diverse geographical regions to support the analysis of dental AI applications worldwide. It includes all languages, patient demographics, and dental and maxillofacial imaging modalities.

### Data Sources

This study selected data sources from academic databases, prints, collaborative platforms, and data repositories, prioritizing prominent AI development resources (Chrimes and Kim 2022). The selected databases included Google Datasets, OpenDataLab CN, Zenodo, Mendeley, figshare, OSF, arXiv, IEEE, medRxiv, GitHub, Kaggle, and the Grand Challenge website, focusing on data and articles from 2020 to 2023. Targeted PubMed searches were also conducted for dental AI research articles published between 2011 and January 2024, as potential dataset references (database details: Appendix Table 1).

### Search Strategy

We searched multiple platforms to identify datasets for AI applications in dental imaging (intraoral, periapical, retroalveolar, intraoral, bite wing, cone-beam computed tomography [CBCT], cephalometric and extraoral radiography). We used keywords and MeSH terms related to dentistry, AI, machine learning, and deep learning. A team of 4 protocol investigators, including the lead author, developed and executed the search strategy. Six investigators, including the lead author, performed duplicate searches followed by a final review by the lead author. Details of the search strategy are provided in Appendix Table 2.

### Inclusion and Exclusion Criteria

Datasets were included if they met the following criteria: publicly accessible or accessible via registration with a minimum of 50 clinical or radiographic dental and maxillofacial images, with or without annotations. No geographical, language, patient demographics, or dental or maxillofacial imaging modality was considered.

Datasets were excluded if they contained fewer than 50 images, focused on nondental or nonmaxillofacial data, comprised only text or numerical data, or were only available “upon request,” as the evidence suggests extremely limited author responsiveness (Gabelica et al. 2022). Two investigators reviewed each potential dataset. Datasets with agreement between 2 reviewers were also included. A third investigator (S.E.U.) resolved any disagreements.

### Study Size

We expect to use all records returned by our search that meet the inclusion criteria.

### Data Extraction

Three online training sessions were conducted for all reviewers to ensure consistency in the data-extraction process. These sessions emphasized extracting forms, defining data elements, and addressing potential discrepancies. Reviewers used exemplary datasets before beginning the formal extraction process. Variables, such as dataset characteristics, ethics, and image details, were extracted. Six researchers performed data extraction, and each dataset was independently assessed in duplicate. To ensure no IP restrictions, the assessments were conducted from different geographical locations, including Latvia, India, Chile, Iran, and Poland, as well as from VPNs in China, Egypt, Canada, and Australia. A single investigator confirmed the results. Each researcher received the dataset URL and used a custom Zoho Survey form designed to capture the data and metadata of the dataset, including:

- General dataset characteristics: Year of publication, research focus, associated publications (including DOI), country of origin, and data collection period.
- Imaging: Source of acquisition, reason for acquisition, and imaging modalities used.
- Technical specifications: Equipment, dataset license, and image processing. If present, licenses determine the terms of use of the dataset, ensure compliance with copyright and intellectual property rights, and enable legal sharing and reuse.
- Ethics: Presence of ethical approval, informed consent practices, and patient inclusion and exclusion criteria.
- Number of patients and images included.
- Annotations: We extracted all potential annotations such as labels or segmentations. Segmentation identifies and demarcates specific anatomical regions or dental lesions in images, enabling a detailed analysis of AI algorithm development in dentistry. Labeling assigns designations to segmented areas, such as anatomical region names or dental condition presence, improves data organization and interpretation, and aids AI model training in recognizing and classifying dental structures and conditions. Ground-truth methods are used to establish the accuracy of annotations, whereas anonymization strategies are used to protect sensitive information in the data.
- Annotator characteristics: (If annotations are present) Number of annotators, calibration methods, experience, and strategies for addressing disagreement.
- Demographics: Reported gender ratio and ethnicity of participants.
- The study protocol (10.17605/OSF.IO/HUZ72) defined other variables, such as the area of interest (oral pathology, cariology, etc.).

For conflicting information (e.g., a discrepancy between the image count stated in the paper and the dataset itself), the dataset repository was considered as the definitive source. The data-extraction codebook is presented in Appendix Table 3.

### Dataset Quality according to FAIR Principles

The FAIR principles, referring to the Findability, Accessibility, Interoperability, and Reusability of digital assets (Wilkinson et al. 2016), were evaluated for each selected dataset using the 41 FAIRsFAIR Data Object Assessment Metrics version 0.5 (Devaraju and Huber 2021) following the methods described previously (Uribe et al. 2022). Findability involves assigning unique and persistent identifiers to data, accessibility ensures data retrieval via standardized protocols, interoperability uses standardized languages and vocabularies for data integration, and reusability requires rich metadata for data reuse in various contexts (Wilkinson et al. 2016).

### Bias Minimization

Duplicate data extraction and a third reviewer were used to minimize potential biases.

### Data Synthesis and Analysis

The unit of analysis was a dataset containing missing data included in the analyses. We aimed to extract the most comprehensive information possible from each dataset to characterize their key properties, focusing on the distributions and central tendencies. Primary outcomes included dataset availability, size, and descriptive attributes, while secondary outcomes were centered on metadata, licensing, and adherence to FAIR principles. R Software (v4.1.2) (R Core Team 2021) was used to generate summary tables, descriptive statistics, and visualizations to explore the dataset characteristics thoroughly.

## Results

**Figure 1.**
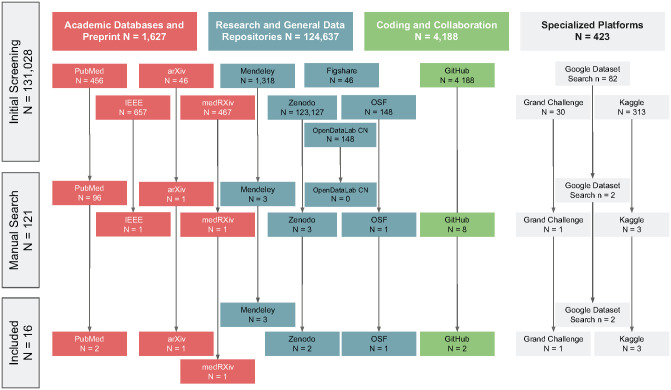
Flowchart of dataset selection.

A total of 131,028 records were identified from the various databases. After the initial screening, 121 records remained in the study. Sixteen unique dental imaging datasets, obtained from various repositories, were included. The most common sources were Kaggle (18.8%), GitHub Google Datasets, Mendeley, PubMed, Zenodo (12.5% each), Grand-Challenge, OSF, and arXiv with 6.2% each. The results of the dataset search are shown in Figure 1.

The number of publicly available datasets has increased over time, with 1 dataset (6.2%) published in 2020, 2 (12.5%) in 2021, 6 (37.5%) in 2022, and 7 (43.8%) in 2023. Most of the datasets (68.8%) were associated with scholarly publications.

The primary research areas identified in the datasets were tooth or lesion segmentation (62.5%) and labeling (56.2%).

Panoramic radiographs were the most common imaging modality (58.8%), followed by CBCT and intraoral photographs (11.8% each), and cephalometric radiographs, intraoral 3-dimensional (3D) scans, and periapical radiographs with 5.9% each.

The datasets varied in terms of the number of images provided, with a mean (standard deviation) of 595 (790) panoramic radiographs, 252 (241) intraoral radiographs, 278 (156) CBCT, and 945 (731) for 3D intraoral scans and intraoral photos.

**Figure 2.**
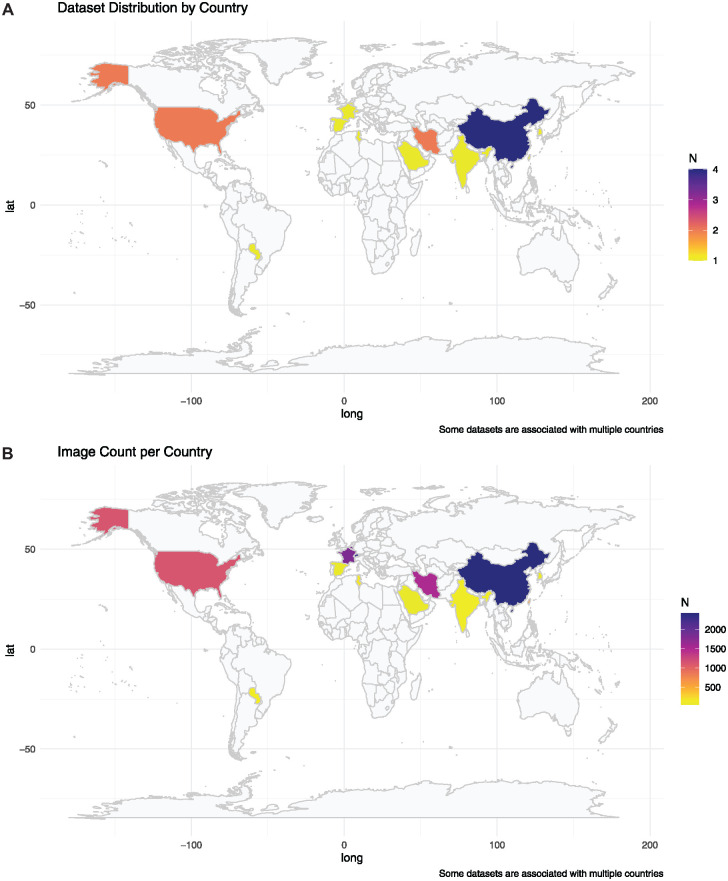
Global density distribution of datasets and images available for dental imaging artificial intelligence.

China contributed the most (2,413), followed by Switzerland (2,332), Belgium, France (1,800 each), Iran (1,504), the United States (1,117), Taiwan (600), Tunisia (180), Paraguay (135), and India (131). Saudi Arabia, South Korea, and Spain provided datasets of 50 images each. Some images were shared between countries, contributing to the overall dataset total. Figure 2 shows the global density distribution of the datasets and number of images by country.

**Figure 3.**
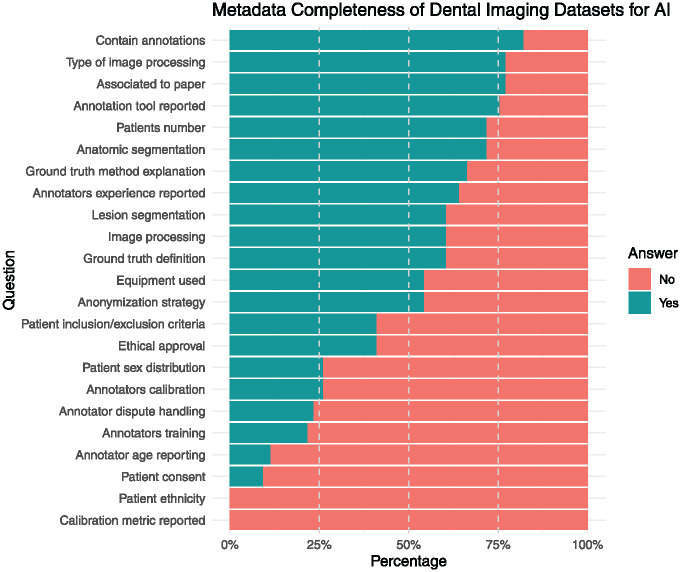
Metadata completeness of dental imaging datasets.

Most of the datasets contained annotations (75%), had an associated scientific publication reporting the type of image processing used (68.8%), and reported anatomic segmentation and the number of patients included (62.5%). However, fewer than half of the datasets reported ethical approval (31.2%), patient sex distribution (18.8%), anonymization strategy (43.8%), and patient inclusion/exclusion criteria (31.2%). Metadata reporting was inconsistent across datasets. While critical metadata elements allowing for external validation, such as the ground-truth definition (56.2%), were frequently included, other metadata critical for ethical considerations and reproducibility, such as those related to ethical considerations and annotations, have been inconsistently reported. Annotator information was provided in 53.8% of the datasets, 18.8% provided annotator calibration details, and 16.7% explained how disagreements were resolved. (Figure 3). Patient consent was reported in 5.9%.

An analysis of the dataset licenses revealed that most datasets (56.3%) did not specify a license. The Creative Commons Attribution Non-Commercial 4.0 (CC BY-NC 4.0), Creative Commons Attribution ShareAlike 4.0 (CC BY-SA 4.0), and Creative Commons Attribution 4.0 (CC BY 4.0) were the most common license types. One dataset was found to have a PhysioNet-restricted health-data license.

Ground-truth determination in the datasets varies: expert decision (3 datasets), majority vote (2 datasets), not determined (3 datasets), and not described (8 datasets). Expert decision relies on specialists’ judgment, while majority vote takes the consensus of multiple annotators. The absence of ground-truth determination or documentation highlights a gap in the datasets.

The datasets contain pixel-level annotations in 6, label annotations in 6, and box annotations in 2 datasets. Pixel-level annotations provide detailed, pixel-specific information; label annotations assign categorical labels to regions or entire images; and box annotations use bounding boxes to identify and classify areas of interest.

The analyzed datasets showed diverse characteristics and focuses. Some datasets, such as those from the Grand Challenge, aim to improve cephalometric landmark detection precision, whereas others, such as the Tongue datasets, focus on specific dental structures. The DENTEX dataset includes 23,999 annotated teeth and was used for the 3DTeethSeg 2022 challenge. Another dataset, Panoramic Dental X-rays With Segmented Mandibles, consists of 232 images with pixel-level instance segmentation annotations for mandibles. Several datasets contained panoramic images, some of which were cropped or low quality. One dataset provides a multimodal approach incorporating CBCT (389), panoramic radiographs (12), and intraoral images generated from CBCT (240) of 389 patients, making it the most comprehensive multimodal dataset.

Most of the data were acquired from dental clinics (62.5%), followed by university dental school clinics (18.75%), hospitals (6.25%), schools (6.25%), and undisclosed sources (12.5%). The reasons for image acquisition were primarily dental diagnosis (50%), followed by research purposes (31.25%), benchmarking (6.25%), diagnostic investigations (6.25%), and oral disease diagnosis (6.25%), with 12.5% of the datasets not specifying the reason for obtaining imagery. Table 1 presents the detailed characteristics of the included datasets, while Appendix Table 4 contains the results of the excluded items.

**Table 1.** Dataset Characteristics for Dental Imaging Artificial Intelligence Applications.

Dataset Name	Database	Dataset URL	Year	What Are the Main Areas of the Dataset/Research? (Select All That Apply)	Country	DOI of the Associated Publication	Source of Data Acquisition	Reason for Image Acquisition
DENTEX Panoramic	Zenodo	https://zenodo.org/records/7812323	2023	Oral surgery	Switzerland	10.48550/arXiv.2305.19112 and 10.48550/arXiv.2303.06500	Dental clinics	Dental diagnosis
CTooth dataset	arXiv	https://www.kaggle.com/datasets/weiweicui/ctooth-dataset	2022	Teeth segmentation, teeth labeling	China	10.1007/978-3-031-17027-0_7	Dental clinics	Dental diagnosis, research
Panoramic Dental X-rays With Segmented Mandibles	Mendeley	https://data.mendeley.com/datasets/hxt48yk462/2	2020	Teeth segmentation, teeth labeling	Iran	10.1117/1.JMI.2.4.044003	Dental clinics	Dental diagnosis, research
Dental radiography	Kaggle	https://www.kaggle.com/datasets/imtkaggleteam/dental-radiography	2023	Teeth segmentation, teeth labeling	Iran		Dental clinic	Dental diagnosis, research
Panoramic-Caries-Segmentation	Github	https://github.com/Zzz512/MLUA	2023	Caries	China	10.1016/j.neucom.2023.03.069	Not described	Not described
TK_Tooth_Number_Code	Github	https://github.com/tanjidakabir/TK_Tooth_Number_Code	2022	Teeth segmentation, teeth labeling			Not described	Not described
CL Detection	Grand-challenge	https://cl-detection2023.grand-challenge.org/	2023	Cehalometric	Taiwan		Dental clinics	Benchmarking
Tufts Panoramic Dataset	PubMed	https://tdd.ece.tufts.edu/	2021	Caries, oral pathology, endodontics, teeth segmentation	United States	10.1109/JBHI.2021.3117575	University dental school clinic	Diagnostic investigation
3DTeethSeg22_challenge ToothFairy or Teeth3DS	OSF	https://osf.io/xctdy/	2022	Teeth segmentation, teeth labeling	France, Belgium	10.48550/arXiv.2210.06094	Dental clinics	Dental diagnosis
v7labs	Google Datasets	https://www.v7labs.com/open-datasets/panoramic-dental	2023	Teeth segmentation, teeth labeling	United States		Dental clinics	Dental diagnosis, research
tooth-marked-tongue	Kaggle	https://www.kaggle.com/datasets/clearhanhui/biyesheji	2022	Oral pathology	China	10.3390/diagnostics12102451	Other—schools	Dental diagnosis
Oral Cancer (Lips and Tongue) images	Kaggle	https://www.kaggle.com/datasets/shivam17299/oral-cancer-lips-and-tongue-images/data	2022	Oral pathology	India		Hospitals	Diagnostic for oral diseases
Pulp Exposure	PubMed	https://doi.org/10.6084/m9.figshare.23930368.v1	2023	Caries, endodontics	Saudi Arabia, Spain, Korea	10.1186/s12903-023-03251-0	Dental clinics, university dental school clinic	Not described
Panoramic-Paraguay	Zenodo	https://zenodo.org/records/4457648	2021	Teeth segmentation, teeth labeling	Paraguay	10.3390/s21093110	University dental school clinic	Not described
Panoramic Dental Xray Dataset	Mendeley	https://data.mendeley.com/datasets/73n3kz2k4k/2	2023	Teeth segmentation, teeth labeling	Tunisia	10.1007/s11042-023-17568-z	Dental clinics	Dental diagnosis, research
PhysioNet Multimodal	Google Datasets	https://physionet.org/content/multimodal-dental-dataset/1.0.0/	2022	Teeth segmentation, teeth labeling	China	10.13026/s5z3-2766.	Dental clinics	Dental diagnosis, research
Dataset Name	Imaging Modality	Equipment Detail	License Type of the Dataset	Female %	Annotators	Annotation Type	Annotation Software	How Was Ground Truth Determined?
DENTEX Panoramic	Panoramic radiographs	VistaPano S X-ray unit (Durr Dental, Germany)	No license specified		Not specified	Label		Not described
CTooth dataset	Cone-beam computed tomography (CBCT)	Op300, Instrumentarium, Finland	CC BY-NC 4.0		Yes	Label	IITKSNAP	Expert decision
Panoramic Dental X-rays With Segmented Mandibles	Panoramic radiographs	Soredex CranexD digital panoramic X-ray unit	CC BY-NC 3.0		2	Pixel level		Not described
Dental Radiography	Panoramic radiographs		CC BY-SA 4.0		Not specified			No
Panoramic-Caries-Segmentation	Panoramic radiographs		No license specified		5	Pixel level		Not described
TK_Tooth_Number_Code	Panoramic radiographs		No license specified		Not specified	Label		Not described
CL Detection	Cephalometric radiographs		No License Specified		Not specified	Pixel level		Not described
Tufts Panoramic Dataset	Panoramic radiographs	OP100 Orthopantomograph (Instrumentarium Imaging/Kavo Kerr) and Plammeca Promax 2D	No license specified		2	Label		Expert decision
3DTeethSeg22_challenge ToothFairy or Teeth3DS	Intraoral 3D scans	Primescan, Trios3, iTero Element 2 Plus	CC BY-NC-ND 4.0	50	Not specified	Pixel level	Custom tool mentioned	Expert decision
v7labs	Panoramic radiographs		No license specified		No	Pixel level		No
tooth-marked-tongue	Intraoral photograph	Canon Eos 700d	No license specified		5	Label	Labelme, Labelimg	Majority vote
Oral Cancer (Lips and Tongue) images	Intraoral photograph		Copyright the authors		Not specified	Label		Not described
Pulp Exposure	Intraoral radiographs		No license specified		10	Box		Majority vote
Panoramic-Paraguay	Panoramic radiographs		No license specified	60	Not specified			Not described
Panoramic Dental Xray Dataset	Panoramic radiographs		CC BY 4.0		2	Pixel level	VGG Image Annotator	Not described
PhysioNet Multimodal	CBCT, panoramic radiographs		PhysioNet Restricted Health Data License 1.5.0	56	13	Box		No
Dataset Name			Patients	Images	Images: Panoramic	Images: Intraoral Radiographs	Images: CBCT	Images: Other
DENTEX Panoramic				2,332	2,332			
CTooth dataset			22	168			168	
Panoramic Dental X-rays With Segmented Mandibles			232	232	232			
Dental Radiography			1,272	1,272	1,272			
Panoramic-Caries-Segmentation				75	75			
TK_Tooth_Number_Code				188		188		
CL Detection			600	600				600
Tufts Panoramic Dataset				1,000	1,000			
3DTeethSeg22_challenge ToothFairy or Teeth3DS			900	1,800				1,800
v7labs			117	117	117			
tooth-marked-tongue			462	1,250				1,250
Oral Cancer (Lips and Tongue) images			No	131				131
Pulp Exposure				50		50		
Panoramic-Paraguay			135	135	135			
Panoramic Dental Xray Dataset			180	180	180			
PhysioNet Multimodal			389	920	12	519	389	
Dataset Name		URL Active	Registration Required	Files Format	Images and Resolutions	Image Quality	Image Manipulation	Contains Additional Information
DENTEX Panoramic		Yes	No	PNG	Panoramic images, 2,048 × 989, 3,118 × 1,250, 2,650 × 1,150, 2,850 × 1,250, 2,700 × 1,200, 2,850 × 1,200, 2,950 × 1,330	Raw images	No	Yes, Readme file, training and validation datasets
CTooth dataset		Yes	Yes	dcm	DICOM	Raw images	No	No
Panoramic Dental X-rays With Segmented Mandibles		Yes	No	PNG	Panoramic images, 2,048 × 989, 3,118 × 1,250, 2,650 × 1,150, 2,850 × 1,250, 2,700 × 1,200, 2,850 × 1,200, 2,950 × 1,330	Cropped	Cropped	No
Dental Radiography		Yes	No	JPEG: .jpg, .jpeg, .jfif, .pjpeg, .pjp	Panoramic images, 256 × 512 px	Cropped	Cropped	Yes, an Excel sheet (named: _annotations.csv) with image size and description of each image
Panoramic-Caries-Segmentation		Yes	No	PNG	Panoramic images, 1,435 × 2,943	Raw images	Cropped	Yes, Readme file
TK_Tooth_Number_Code		Yes	No	PNG	Cropped panoramic images, 250 × 152	Raw images	Cropped	Yes, read me file, model description, mask file, and several xlsx files to describe the data
CL Detection		Yes	Yes	JPEG: .jpg, .jpeg, .jfif, .pjpeg, .pjp	Cephalometric, 2048 × 1555 and 1804 × 2148	Raw images	No	No
Tufts Panoramic Dataset		Yes	Yes	JPEG: .jpg, .jpeg, .jfif, .pjpeg, .pjp	Panoramic images, 840 × 1,615	Raw images	No	No
3DTeethSeg22_challenge ToothFairy or Teeth3DS		Yes	No	dcm	DICOM	Raw images	No	Yes, annotations included
v7labs		Yes	No	PNG	Panoramic images, 1,250 × 3,122 px	Raw images	No	No
tooth-marked-tongue		Yes	No	PNG	Photos, 861 × 755 (height: 861 px, width: 755 px)	Raw images	No	No
Oral Cancer (Lips and Tongue) images		Yes	No	JPEG: .jpg, .jpeg, .jfif, .pjpeg, .pjp	Photos, 825 × 1,100 px	Raw images	No	No
Pulp Exposure		Yes	No	JPEG: .jpg, .jpeg, .jfif, .pjpeg, .pjp	Periapical, 1,402 × 1,876	Raw images	No	No
Panoramic-Paraguay		Yes	No	JPEG: .jpg, .jpeg, .jfif, .pjpeg, .pjp	Panoramic images, 1,024 × 2,041 px	Raw images	No	No
Panoramic Dental Xray Dataset		Yes	No	JPEG: .jpg, .jpeg, .jfif, .pjpeg, .pjp	Panoramic images, 1,464 × 2,964 px	Raw images	No	No
PhysioNet Multimodal		Yes	Yes	dcm	DICOM	Raw images	No	Yes, LICENSE.txt and Excel files

From the machine’s perspective, the quality of the dental imaging datasets varies in terms of their compliance with the FAIR principles. Panoramic radiographs and CBCT datasets have advanced FAIRness levels, with scores of 75 and 64, respectively. Intraoral radiographs have a moderate FAIRness level of 75, while intraoral photographs have a moderate level of 60. However, some datasets have lower FAIRness levels, such as cephalometric radiographs and intraoral 3D scans, both with initial levels of 27 and 41, respectively. The characteristics of these datasets vary according to the FAIR principles, as shown in Table 2. For instance, the FAIRness level of panoramic radiographs ranges from initial to advanced, while CBCT datasets have consistently advanced FAIRness levels. Intraoral radiographs and photographs have moderate FAIRness levels, whereas cephalometric radiographs and intraoral 3D scans have initial FAIRness levels.

**Table 2.** Summary Characteristics of Dental Imaging Datasets by Imaging Modality.

Imaging Modality	Datasets	Images	Country	Findability (max 7)	Accessibility (max 3)	Interoperability (max 4)	Reusability (max 10)
Cone-beam computed tomography	2	557	CHN	3.5	1.7	2.3	3.3
Intraoral radiographs	3	757	ESP, KOR SAU	7	2	3	6
Panoramic radiographs	9	5355	CHE, CHN, IRN PRY, TUN, USA	3.8	1.8	2	3.8
Other	4	3781	BEL, CHN, FRA, IND TWN	3.5	1.8	1.6	4.2

BEL, Belgium; CHE, Switzerland; CHN, China; ESP, Spain; FRA, France; IND, India; IRN, Iran; KOR, South Korea; PRY, Paraguay; SAU, Saudi Arabia; TUN, Tunisia; TWN, Taiwan; USA, United States of America.

## Discussion

This study provides the first comprehensive overview of publicly available dental imaging datasets that are suitable for AI development. We identified 16 unique datasets comprising 10,450 images with significant variations in size, imaging modalities, pathologies, and metadata completeness.

This study expands the geographic representation of dental imaging datasets, with data from 13 countries and 44.6% of the global population. This represents a significant advance over previous research, which relied primarily on medical datasets heavily centered in the United States and China (Celi et al. 2022). Although progress has been made, there are still significant gaps in representation, with Oceania having no datasets and Africa having only one.

To develop truly generalizable AI models, dataset diversification is critical to ensure that models capture global variations in oral health needs and disease presentations.

Compared with a previous study from medical radiology (Venkatesh et al. 2022), our search covered all available sources and not just the data associated with publications. Although we found a similar number of available datasets in dentistry (16) compared with medicine (12), the number of patients and images in medical radiology significantly surpasses that of dental imaging, with 10,450 dental images identified in our study versus more than 11 million patients and 62 million images in medical radiology (Mongan and Halabi 2023). Notably, even within oral medicine, the number of datasets and images varied widely; a recent publication found only 1 dataset focusing on oral cancer (Sengupta et al. 2022)

Panoramic radiographs remain the predominant imaging modality in datasets, likely owing to their widespread availability and diagnostic value. Moreover, anatomical image segmentation was the focus of the present datasets when assessing the provided annotations. A wider set of annotations and cross-annotations with clinical and omic data is needed to unlock AI’s full potential tasks, such as disease detection, anatomical segmentation, and labeling. Typically, the same images used for annotation served as the ground-truth source. However, in clinical situations, imaging is often complemented with other clinical information and validated using established reference tests such as laboratory studies. Data must be cross-annotated to overcome human examiners’ limitations and enhance the power of clinical images. By annotating images with cross-data, such as lab studies, AI can extract or detect patterns humans may find difficult to perceive, ultimately improving dentistry’s diagnostic accuracy and treatment-planning capabilities. Moreover, future studies should aim to provide a more comprehensive multimodal dataset for the included patients, that is, to consider available intraoral photographs, CBCT, or 3D intraoral scans, as multimodal learning will likely allow for more precise modeling (Liu et al. 2023),

The datasets exhibited significant variation in quality regarding characteristics, metadata, licensing, and adherence to FAIR principles. While crucial metadata for AI development (annotations, image processing methods, and reference test definitions) were often included, metadata essential for reproducibility and ethical considerations were less frequent. This inconsistency raises concerns about dataset reproducibility and ethical soundness. The FAIR principle analysis revealed intraoral radiographs as the most findable modality, whereas CBCT datasets scored the lowest overall. This suggests uneven quality and usability across the imaging types. Interestingly, most datasets in our study showed moderate (37.5%) to advanced (25%) FAIRness, in contrast to prior analyses of dental research data reporting low FAIRness (Uribe et al. 2022). This discrepancy may arise because we focused on datasets designed for automated processing and searched beyond dental publications. A major limitation is the frequent absence of specified licenses, which creates ambiguity regarding data reusability and sharing rights.

Furthermore, inconsistent reporting of ground-truth establishment methods reduces transparency, affecting the reliability of the AI models developed from these datasets (Dumitrache et al. 2021). Variations in ground truths and the lack of standardized reference tests introduce further uncertainty (Sylolypavan et al. 2023), impacting model performance evaluation. Addressing these ground-truth challenges is essential for the development of reliable and valid AI models in dentistry.

Accessing large-scale dental imaging data for AI models is challenging owing to governance, costs, time lags, data quality, and lack of labeling (Celi et al. 2022). Public datasets can help overcome these barriers (Celi et al. 2022). We propose establishing a centralized, searchable repository following FAIR principles, promoting dataset diversity, incentivizing data sharing through journal policies (Schwendicke et al. 2022), and developing standardized metadata guidelines from dental AI datasets such as “Data Cards” (Pushkarna et al. 2022). Data Cards provide structured summaries of ML dataset information, addressing standardization gaps. Solutions such as Croissant Format Specification (Benjelloun et al. 2024) can add missing metadata programmatically to existing datasets. Medical AI Data for All (MAIDA) (Saenz et al. 2024) and the Global Initiative on AI for Health (GI-AI4H) (World Health Organization 2023) are notable initiatives that address dataset availability for AI in health care, including dentistry. By focusing on dataset diversity, data sharing, and standardized metadata guidelines, the AI community can improve oral health worldwide.

Our study had several limitations. We searched publicly available datasets only from selected databases, potentially overlooking some datasets only accessible through institutional or research collaborations. The dynamic nature of dataset availability means that some findings may not remain accessible. We did not evaluate dataset quality or internal biases, which are crucial for assessing the suitability and reliability of datasets for AI applications. Regular updates and future research should address these issues to ensure the accuracy and relevance of this resource.

## Conclusion

This study provides the first comprehensive assessment of dental imaging datasets in AI, revealing a scarcity of publicly available data and inconsistent metadata reporting. To promote the development of robust, equitable, and generalizable AI tools for dental diagnostics, treatment, and research, efforts are needed to address data scarcity, increase diversity, mandate metadata completeness, and ensure FAIRness in AI dental imaging research.

## Author Contributions

S.E. Uribe, contributed to conception and design, data acquisition, analysis, and interpretation, drafted and critically revised the manuscript; J. Issa, F. Sohrabniya, A. Denny, N.N. Kim, A.F. Dayo, A. Chaurasia, A. Sofi-Mahmudi, M. Büttner, contributed to data acquisition, critically revised the manuscript; F. Schwendicke, contributed to design, contributed to data interpretation, drafted and critically revised the manuscript. All authors gave final approval and agree to be accountable for all aspects of the work.

## Supplemental Material

sj-docx-1-jdr-10.1177_00220345241272052 – Supplemental material for Publicly Available Dental Image Datasets for Artificial Intelligence Supplemental material, sj-docx-1-jdr-10.1177_00220345241272052 for Publicly Available Dental Image Datasets for Artificial Intelligence by S.E. Uribe, J. Issa, F. Sohrabniya, A. Denny, N.N. Kim, A.F. Dayo, A. Chaurasia, A. Sofi-Mahmudi, M. Büttner and F. Schwendicke in Journal of Dental Research
