# Supplementary material for: Publicly Available Dental Image Datasets for Artificial Intelligence
Source: J Dent Res. 2024 Oct 18;103(13):1365–74. doi: 10.1177/00220345241272052 (PMC11633071; doi:10.1177/00220345241272052)
Supplement: sj-docx-1-jdr-10.1177_00220345241272052 – Supplemental material for Publicly Available Dental Image Datasets for Artificial Intelligence [file sj-docx-1-jdr-10.1177_00220345241272052.docx]

# APPENDIX

## Appendix Table 1: Databases searched.

| **Site** | **Description** | **Company/Owner/Organization** |
| --- | --- | --- |
| arXiv | A repository of electronic preprints of scientific papers in various fields | Cornell University |
| Google Dataset Search | A search engine for finding publicly available datasets hosted on various platforms | Google |
| Grand Challenge | A a comprehensive platform for the end-to-end development of machine learning solutions in biomedical imaging | Radboud University Medical Center |
| figshare | A repository for sharing and citing research data | Digital Science |
| GitHub | A platform for version control and collaboration on software development projects | Microsoft |
| IEEE Xplore | A digital library providing access to IEEE journals, transactions, magazines, and conference papers | IEEE |
| Open Science Framework | A platform for managing and sharing research projects and data | Center for Open Science |
| Kaggle | A platform for data science competitions and collaboration | Google |
| medRxiv | A preprint server for health sciences research | Cold Spring Harbor Lab Press |
| Mendeley | A reference manager and academic social network for researchers | Elsevier |
| PubMed | A free search engine accessing primarily the MEDLINE database of references and abstracts on life sciences and biomedical topics | U.S. National Library of Medicine (NLM) |
| Zenodo | A digital repository for sharing, preserving, and citing research outputs | CERN |
| OpenDataLab CN | Is a chinese platform that provides access to numerous open-source datasets, | Ministry of Industry and Information Technology (MIIT) of the People's Republic of China |

## Appendix Table 2. Database Search Strategies.

| **Database** | **Comments** | **Hits** | **Search strategy** |
| --- | --- | --- | --- |
| PubMed | #1 ("Dentistry"[MeSH Terms] AND ("Artificial Intelligence"[MeSH Terms] OR "Machine Learning"[MeSH Terms] OR "neural networks, computer"[MeSH Terms])) AND (2020:2022[pdat] | 485 | ("Dentistry"[MeSH Terms] AND ("Artificial Intelligence"[MeSH Terms] OR "Machine Learning"[MeSH Terms] OR "neural networks, computer"[MeSH Terms])) AND (2020:2023[pdat]) |
| PubMed | #2 Extended PubMed search with Text Words and MeSH Terms: T | 1007 | ("dentistry"[MeSH Terms] AND ("Artificial Intelligence"[MeSH Terms] OR ("Artificial Intelligence"[Text Word] OR "Machine Learning"[Text Word] OR "Artificial Learning"[Text Word] OR "Bayesian Learning"[Text Word] OR "Deep Learning"[Text Word] OR "Knowledge Representation"[Text Word] OR "neural network*"[Text Word] OR "probabilistic network*"[Text Word] OR "Statistical Learning"[Text Word] OR "support vector machine*"[Text Word] OR "generalized linear model*"[Text Word] OR "naive bayes*"[Text Word] OR "ensemble method*"[Text Word] OR "neural network model*"[Text Word] OR "decision tree*"[Text Word] OR "proportional hazards model*"[Text Word] OR "Long short term memory"[Text Word] OR "Natural language processing"[Text Word] OR "Speech recognition"[Text Word] OR "Robotics"[Text Word] OR "sensor*"[Text Word] OR "Gamification"[Text Word] OR "Automated planning"[Text Word]))) AND (2020:2023[pdat]) |
| PubMed | #3 General AI-related terms search: | 266435 | "artificial intelligence"[All Fields] OR "deep learning"[All Fields] OR "machine learning"[All Fields] OR "neural network"[All Fields] |
| PubMed | #4 Specific AI models and techniques | 107464 | ("Random Forest"[All Fields] AND "Ensemble Machine Learning"[All Fields]) OR "Ridge Regression"[All Fields] OR "Neighborhood Component Analysis"[All Fields] OR "Discriminant Analysis"[All Fields] OR "Linear Discriminant Analysis"[All Fields] OR "Recursive Feature Elimination"[All Fields] OR "Fuzzy Logic Analysis"[All Fields] OR "Naive Bayes"[All Fields] OR "Hierarchical Clustering"[All Fields] OR "Local Active Contour"[All Fields] OR "k-Nearest Neighbor"[All Fields] OR "Decision Tree"[All Fields] OR "Fuzzy C-means Clustering"[All Fields] OR "Least Absolute Shrinkage and Selection Operator"[All Fields] OR "Support Vector Machine"[All Fields] OR "Support Vector Regression"[All Fields] OR "Linear Kernel SVM"[All Fields] OR "Artificial Neural Network"[All Fields] OR "Gradient Boosting"[All Fields] OR (("multiple"[All Fields] OR "multiples"[All Fields]) AND ("kernel"[All Fields] OR "kernel s"[All Fields] OR "kernelization"[All Fields] OR "kernelized"[All Fields] OR "kernels"[All Fields]) AND ("machine learning"[MeSH Terms] OR ("machine"[All Fields] AND "learning"[All Fields]) OR "machine learning"[All Fields])) OR "Adaptive Neuro-Fuzzy Inference System"[All Fields] OR "Generative Adversarial Network"[All Fields] |
| PubMed | #5 Specific AI models and techniques 2 | 202 | ("Convolutional Neural Networks"[All Fields] OR "Residual Networks"[All Fields] OR "Autoencoders"[All Fields] OR "Recurrent Neural Networks"[All Fields] ) AND ("teeth s"[All Fields] OR "teeths"[All Fields] OR "tooth"[MeSH Terms] OR "tooth"[All Fields] OR "teeth"[All Fields] OR "tooth s"[All Fields] OR "tooths"[All Fields] OR ("teeth s"[All Fields] OR "teeths"[All Fields] OR "tooth"[MeSH Terms] OR "tooth"[All Fields] OR "teeth"[All Fields] OR "tooth s"[All Fields] OR "tooths"[All Fields]) OR "maxillofacial"[All Fields] OR "temporomandibular"[All Fields] OR ("buccal"[All Fields] OR "buccally"[All Fields]) OR ("carie"[All Fields] OR "dental caries"[MeSH Terms] OR ("dental"[All Fields] AND "caries"[All Fields]) OR "dental caries"[All Fields] OR "caries"[All Fields]) OR ("periodontal"[All Fields] OR "periodontally"[All Fields] OR "periodontically"[All Fields] OR "periodontics"[MeSH Terms] OR "periodontics"[All Fields] OR "periodontic"[All Fields] OR "periodontitis"[MeSH Terms] OR "periodontitis"[All Fields] OR "periodontitides"[All Fields]) OR ("orthodontal"[All Fields] OR "orthodontic"[All Fields] OR "orthodontical"[All Fields] OR "orthodontically"[All Fields] OR "orthodontics"[MeSH Terms] OR "orthodontics"[All Fields])) AND (2020:2023[pdat]) |
| PubMed | #6 Combining (1+2+3+4+5) with OR | 2503 | ("Dentistry"[MeSH Terms] AND ("Artificial Intelligence"[MeSH Terms] OR "Machine Learning"[MeSH Terms] OR "neural networks, computer"[MeSH Terms])) AND (2020:2023[pdat]) OR ("dentistry"[MeSH Terms] AND ("Artificial Intelligence"[MeSH Terms] OR ("Artificial Intelligence"[Text Word] OR "Machine Learning"[Text Word] OR "Artificial Learning"[Text Word] OR "Bayesian Learning"[Text Word] OR "Deep Learning"[Text Word] OR "Knowledge Representation"[Text Word] OR "neural network*"[Text Word] OR "probabilistic network*"[Text Word] OR "Statistical Learning"[Text Word] OR "support vector machine*"[Text Word] OR "generalized linear model*"[Text Word] OR "naive bayes*"[Text Word] OR "ensemble method*"[Text Word] OR "neural network model*"[Text Word] OR "decision tree*"[Text Word] OR "proportional hazards model*"[Text Word] OR "Long short term memory"[Text Word] OR "Natural language processing"[Text Word] OR "Speech recognition"[Text Word] OR "Robotics"[Text Word] OR "sensor*"[Text Word] OR "Gamification"[Text Word] OR "Automated planning"[Text Word]))) AND (2020:2023[pdat]) OR "artificial intelligence"[All Fields] OR "deep learning"[All Fields] OR "machine learning"[All Fields] OR "neural network"[All Fields] OR ("Random Forest"[All Fields] AND "Ensemble Machine Learning"[All Fields]) OR "Ridge Regression"[All Fields] OR "Neighborhood Component Analysis"[All Fields] OR "Discriminant Analysis"[All Fields] OR "Linear Discriminant Analysis"[All Fields] OR "Recursive Feature Elimination"[All Fields] OR "Fuzzy Logic Analysis"[All Fields] OR "Naive Bayes"[All Fields] OR "Hierarchical Clustering"[All Fields] OR "Local Active Contour"[All Fields] OR "k-Nearest Neighbor"[All Fields] OR "Decision Tree"[All Fields] OR "Fuzzy C-means Clustering"[All Fields] OR "Least Absolute Shrinkage and Selection Operator"[All Fields] OR "Support Vector Machine"[All Fields] OR "Support Vector Regression"[All Fields] OR "Linear Kernel SVM"[All Fields] OR "Artificial Neural Network"[All Fields] OR "Gradient Boosting"[All Fields] OR (("multiple"[All Fields] OR "multiples"[All Fields]) AND ("kernel"[All Fields] OR "kernel s"[All Fields] OR "kernelization"[All Fields] OR "kernelized"[All Fields] OR "kernels"[All Fields]) AND ("machine learning"[MeSH Terms] OR ("machine"[All Fields] AND "learning"[All Fields]) OR "machine learning"[All Fields])) OR "Adaptive Neuro-Fuzzy Inference System"[All Fields] OR "Generative Adversarial Network"[All Fields] OR ("Convolutional Neural Networks"[All Fields] OR "Residual Networks"[All Fields] OR "Autoencoders"[All Fields] OR "Recurrent Neural Networks"[All Fields] ) AND ("teeth s"[All Fields] OR "teeths"[All Fields] OR "tooth"[MeSH Terms] OR "tooth"[All Fields] OR "teeth"[All Fields] OR "tooth s"[All Fields] OR "tooths"[All Fields] OR ("teeth s"[All Fields] OR "teeths"[All Fields] OR "tooth"[MeSH Terms] OR "tooth"[All Fields] OR "teeth"[All Fields] OR "tooth s"[All Fields] OR "tooths"[All Fields]) OR "maxillofacial"[All Fields] OR "temporomandibular"[All Fields] OR ("buccal"[All Fields] OR "buccally"[All Fields]) OR ("carie"[All Fields] OR "dental caries"[MeSH Terms] OR ("dental"[All Fields] AND "caries"[All Fields]) OR "dental caries"[All Fields] OR "caries"[All Fields]) OR ("periodontal"[All Fields] OR "periodontally"[All Fields] OR "periodontically"[All Fields] OR "periodontics"[MeSH Terms] OR "periodontics"[All Fields] OR "periodontic"[All Fields] OR "periodontitis"[MeSH Terms] OR "periodontitis"[All Fields] OR "periodontitides"[All Fields]) OR ("orthodontal"[All Fields] OR "orthodontic"[All Fields] OR "orthodontical"[All Fields] OR "orthodontically"[All Fields] OR "orthodontics"[MeSH Terms] OR "orthodontics"[All Fields])) AND (2020:2023[pdat]) |
| PubMed | Filtering #6 with dental imaging | 456 | #6 AND ("panoramic radiography" OR "orthopantomography") OR ("intraoral radiography" OR "dental radiographs") OR ("bitewing radiography" OR "bitewing x-rays") OR ("CBCT" OR "cone beam computed tomography") OR ("dental MRI" OR "oral MRI") |
| Google Datasets | Panoramic Dental Xray Dataset | 5 | <https://datasetsearch.research.google.com/search?src=2&query=Panoramic%20Dental%20Xray%20Dataset&docid=L2cvMTFuZ2h5eTY5bg%3D%3D> |
| Google Datasets | Dental Xray Dataset | 75 | <https://datasetsearch.research.google.com/search?src=0&query=Dental%20Xray%20Dataset&docid=L2cvMTFuZ2h5eTY5bg%3D%3D> |
| arXiv | Dentistry AND "Artificial Intelligence" OR "Machine Learning" OR "neural network" OR "deep Learning" | 6 | <https://arxiv.org/search/advanced?advanced=&terms-0-operator=AND&terms-0-term=Dentistry+AND+%22Artificial+Intelligence%22+OR+%22Machine+Learning%22+OR+%22neural+network%22+OR+%22deep+Learning%22&terms-0-field=all&classification-physics_archives=all&classification-include_cross_list=include&date-year=&date-filter_by=date_range&date-from_date=2020&date-to_date=2022&date-date_type=submitted_date&abstracts=show&size=50&order=-announced_date_first> |
| arXiv | dentistry AND panoramic OR periapical OR intraoral OR cbct OR "dental radiograph" OR "dental radiology" AND "Artificial Intelligence" | 35 | <https://arxiv.org/search/advanced?advanced=&terms-0-operator=AND&terms-0-term=dentistry&terms-0-field=all&terms-1-operator=AND&terms-1-term=panoramic&terms-1-field=all&terms-2-operator=OR&terms-2-term=periapical&terms-2-field=all&terms-3-operator=OR&terms-3-term=intraoral&terms-3-field=all&terms-4-operator=OR&terms-4-term=cbct&terms-4-field=all&terms-5-operator=AND&terms-5-term=&terms-5-field=all&terms-6-operator=OR&terms-6-term=%22dental+radiograph%22&terms-6-field=all&terms-7-operator=OR&terms-7-term=%22dental+radiology%22&terms-7-field=all&terms-8-operator=AND&terms-8-term=%22Artificial+Intelligence%22&terms-8-field=all&terms-9-operator=OR&terms-9-term=&terms-9-field=all&classification-computer_science=y&classification-physics_archives=all&classification-include_cross_list=include&date-year=&date-filter_by=date_range&date-from_date=2022&date-to_date=2022&date-date_type=submitted_date&abstracts=show&size=50&order=-announced_date_first> |
| arXiv | dentistry AND dataset | 5 | <https://arxiv.org/search/advanced?advanced=&terms-0-operator=AND&terms-0-term=dentistry&terms-0-field=all&terms-1-operator=AND&terms-1-term=dataset&terms-1-field=all&classification-computer_science=y&classification-physics_archives=all&classification-include_cross_list=include&date-year=&date-filter_by=date_range&date-from_date=2020&date-to_date=2022&date-date_type=submitted_date&abstracts=show&size=50&order=-announced_date_first> |
| medRxiv | "Dentistry AND "Artificial Intelligence" OR "Machine Learning" OR "neural network" OR "deep Learning" " | 94 | <https://www.medrxiv.org/search/Dentistry%252BAND%252B%2522Artificial%252BIntelligence%2522%252BOR%252B%2522Machine%252BLearning%2522%252BOR%252B%2522neural%252Bnetwork%2522%252BOR%252B%2522deep%252BLearning%2522%252B%20numresults%3A75%20sort%3Apublication-date%20direction%3Adescending> |
| medRxiv | "dentistry AND panoramic OR periapical OR cbct OR "dental radiograph" AND "Artificial Intelligence" OR "machine learning"" | 4 | <https://www.medrxiv.org/search/dentistry%252BAND%252Bpanoramic%252BOR%252Bperiapical%252BOR%252Bcbct%252BOR%252B%2522dental%252Bradiograph%2522%252BAND%252B%2522Artificial%252BIntelligence%2522%252BOR%252B%2522machine%252Blearning%2522%20jcode%3Amedrxiv%20numresults%3A10%20sort%3Arelevance-rank%20format_result%3Astandard> |
| medRxiv | "dentistry AND dataset" | 369 | <https://www.medrxiv.org/search/%2522dentistry%252BAND%252Bdataset%2522> |
| IEEE | ("All Metadata":dentistry) AND (("All Metadata":artificial intelligence) OR ("All Metadata":machine learning) OR ("All Metadata":deep learning) OR ("All Metadata":neural network) | 468 | <https://ieeexplore.ieee.org/search/searchresult.jsp?newsearch=true&queryText=(%22All%20Metadata%22:dentistry)%20AND%20((%22All%20Metadata%22:artificial%20intelligence)%20OR%20(%22All%20Metadata%22:machine%20learning)%20OR%20(%22All%20Metadata%22:deep%20learning)%20OR%20(%22All%20Metadata%22:neural%20network))> |
| IEEE | ("All Metadata":oral lesion) AND (("All Metadata":artificial intelligence) OR ("All Metadata":machine learning) OR ("All Metadata":deep learning) OR ("All Metadata":neural network) | 36 | <https://ieeexplore.ieee.org/search/searchresult.jsp?newsearch=true&queryText=(%22All%20Metadata%22:oral%20lesion)%20AND%20((%22All%20Metadata%22:artificial%20intelligence)%20OR%20(%22All%20Metadata%22:machine%20learning)%20OR%20(%22All%20Metadata%22:deep%20learning)%20OR%20(%22All%20Metadata%22:neural%20network))> |
| IEEE | ("All Metadata":dental x-ray) AND (("All Metadata":artificial intelligence) OR ("All Metadata":machine learning) OR ("All Metadata":deep learning) OR ("All Metadata":neural network) | 0 |  |
| IEEE | ("All Metadata":dental) AND ("All Metadata":dataset) | 153 | <https://ieeexplore.ieee.org/search/searchresult.jsp?action=search&newsearch=true&matchBoolean=true&queryText=(%22All%20Metadata%22:dental)%20AND%20(%22All%20Metadata%22:dataset)> |
| Mendeley | "Dentistry" AND "Artificial Intelligence" | 254 | <https://www.mendeley.com/search/?page=1&query=%22Dentistry%22%20AND%20%22Artificial%20Intelligence%22&sortBy=relevance> |
| Mendeley | "Dentistry" AND "Machine Learning" | 126 | <https://www.mendeley.com/search/?page=1&query=%22Dentistry%22%20AND%20%22Machine%20Learning%22&sortBy=relevance> |
| Mendeley | "Dentistry" AND "Neural Network" | 161 | <https://www.mendeley.com/search/?page=1&query=%22Dentistry%22%20AND%20%22Neural%20Network%22&sortBy=relevance> |
| Mendeley | "Dentistry" AND "Artificial intelligence" OR "Artificial learning" OR "Machine learning" OR "Neural Network" OR "Bayesian" | 61 | <https://www.mendeley.com/search/?page=1&query=%22Dentistry%22%20AND%20%22Artificial%20intelligence%22%20OR%20%22Artificial%20learning%22%20OR%20%22Machine%20learning%22%20OR%20%22Neural%20Network%22%20OR%20%22Bayesian%22&sortBy=relevance> |
| Mendeley | "Random forrest" | 476 | <https://www.mendeley.com/search/?page=1&query=Random%20forrest&sortBy=relevance> |
| Mendeley | "Panoramic X-ray dataset" | 54 | <https://www.mendeley.com/search/?page=1&query=Panoramic%20X-ray%20dataset&sortBy=relevance> |
| Mendeley | "Dental Xray Dataset" | 184 | <https://www.mendeley.com/search/?page=1&query=Dental%20X-ray%20dataset&sortBy=relevance> |
| OSF | dental AND dataset | 14 | <https://osf.io/search/?q=dental%20AND%20dataset&page=1> |
| OSF | dental AND ((artificial intelligence) OR (machine learning) OR (deep learning) OR (neural network)) | 103 | <https://osf.io/search/?q=dental%20AND%20((artificial%20intelligence)%20OR%20(machine%20learning)%20OR%20(deep%20learning)%20OR%20(neural%20network))&page=1> |
| OSF | (orthodontics) AND ((artificial intelligence) OR (machine learning) OR (deep learning) OR (neural network)) | 13 | <https://osf.io/search/?q=(orthodontics)%20AND%20((artificial%20intelligence)%20OR%20(machine%20learning)%20OR%20(deep%20learning)%20OR%20(neural%20network))&page=1> |
| OSF | (panoramic) AND ((artificial intelligence) OR (machine learning) OR (deep learning) OR (neural network)) | 11 | <https://osf.io/search/?q=(panoramic)%20AND%20((artificial%20intelligence)%20OR%20(machine%20learning)%20OR%20(deep%20learning)%20OR%20(neural%20network))&page=1> |
| OSF | (CBCT) AND ((artificial intelligence) OR (machine learning) OR (deep learning) OR (neural network)) | 7 | <https://osf.io/search/?q=(CBCT)%20AND%20((artificial%20intelligence)%20OR%20(machine%20learning)%20OR%20(deep%20learning)%20OR%20(neural%20network))&page=1> |
| kaggle | dentistry | 68 | <https://www.kaggle.com/search?q=dentistry+in%3Adatasets> |
| kaggle | dental | 59 | <https://www.kaggle.com/search?q=dental+in%3Adatasets> |
| kaggle | panoramic | 10 | <https://www.kaggle.com/search?q=panoramic+in%3Adatasets> |
| kaggle | cbct | 3 | <https://www.kaggle.com/search?q=cbct+in%3Adatasets> |
| kaggle | oral | 173 | <https://www.kaggle.com/search?q=oral+in%3Adatasets> |
| Grand Challenge | Dental | 2 | <https://grand-challenge.org/challenges/?search=dentistry&educational=unknown&status=&submit=Apply+Filters> |
| Grand Challenge | Dentistry | 2 | <https://grand-challenge.org/challenges/?search=dentistry&educational=unknown&status=&submit=Apply+Filters> |
| Grand Challenge | Oral disease | 2 | <https://grand-challenge.org/challenges/?search=oral&diseases=unknown&status=&submit=Apply+Filters> |
| Grand Challenge | teeth | 1 | <https://grand-challenge.org/challenges/?search=teeth&educational=unknown&status=&submit=Apply+Filters> |
| Grand Challenge | Dental | 2 | <https://grand-challenge.org/challenges/?search=dentistry&educational=unknown&status=&submit=Apply+Filters> |
| Grand Challenge | "dental" AND "artificial intelligence" | 21 | <https://figshare.com/search?q=%22dental%22%20AND%20%22artificial%20intelligence%22&pubPublishDate=2020-01-01%2C2022-12-29> |
| figshare | "oral lesion" AND "artificial intelligence" | 2 | <https://figshare.com/search?q=%22oral%20lesion%22%20AND%20%22artificial%20intelligence%22&pubPublishDate=2020-01-01%2C2022-12-29> |
| figshare | "oral" AND "deep learning" | 21 | <https://figshare.com/search?q=%22oral%22%20AND%20%22deep%20learning%22&pubPublishDate=2020-01-01%2C2022-12-29> |
| figshare | "dental" AND "machine learning" | 12 | <https://figshare.com/search?q=%22dental%22%20AND%20%22machine%20learning%22&pubPublishDate=2020-01-01%2C2022-12-29> |
| figshare | "CBCT" AND "Artificial intelligence" | 3 | <https://figshare.com/search?q=%22CBCT%22%20AND%20%22artificial%20intelligence%22&pubPublishDate=2020-01-01%2C2022-12-29> |
| figshare | "Orthodontics" AND "Artficial intelligence" | 8 | <https://figshare.com/search?q=%22orthodontics%22%20AND%20%22artificial%20intelligence%22&pubPublishDate=2020-01-01%2C2022-12-29> |
| github | Panoramic | 3527 | <https://github.com/search?l=&p=6&q=tooth&ref=advsearch&type=Repositories> |
| github | Dental datasets | 6 | <https://github.com/search?q=dental+datasets+created%3A2020+created%3A2021+created%3A2022&type=Repositories> |
| github | CBCT | 103 | <https://github.com/search?q=CBCT+created%3A2020+created%3A2021+created%3A2022&type=Repositories> |
| github | Oral lesion | 19 | <https://github.com/search?q=oral+lesion+created%3A2020+created%3A2021+created%3A2022&type=Repositories&ref=advsearch&l=&l=> |
| github | Dental AI | 35 | <https://github.com/search?q=dental+AI+created%3A2022+created%3A09+created%3A29+created%3A2020+created%3A2021+created%3A2022-09-29&type=Repositories> |
| github | Oral cancer | 89 | <https://github.com/search?q=oral+cancer+created%3A2022+created%3A09+created%3A29+created%3A2020+created%3A2021+created%3A2022-09-29&type=Repositories> |
| github | Dentistry | 409 | <https://github.com/search?q=dentistry+created%3A2022+created%3A09+created%3A29+created%3A2020+created%3A2021+created%3A2022-09-29&type=Repositories&ref=advsearch&l=&l=> |
| zenodo | publication_date:[2020-01-01 TO 2022-09-29] AND title: (dentistry OR dental) AND (artificial intelligence) OR (machine learning) OR (deep learning) OR (neural network) | 10 | <https://zenodo.org/search?page=1&size=20&q=publication_date:%5B2020-01-01%20TO%202022-09-29%5D%20AND%20title:%20(dentistry%20OR%20dental)%20AND%20(artificial%20intelligence)%20OR%20(machine%20learning)%20OR%20(deep%20learning)%20OR%20(neural%20network)&sort=-publication_date> |
| zenodo | publication_date:[2020-01-01 TO 2022-09-29] AND title: tooth AND (artificial intelligence) OR (machine learning) OR (deep learning) OR (neural network) | 7 | <https://zenodo.org/search?page=1&size=20&q=publication_date:%5B2020-01-01%20TO%202022-09-29%5D%20AND%20title:%20tooth%20AND%20(artificial%20intelligence)%20OR%20(machine%20learning)%20OR%20(deep%20learning)%20OR%20(neural%20network)&sort=-publication_date> |
| zenodo | publication_date:[2020-01-01 TO 2022-09-29] AND title: (oral disease) AND (artificial intelligence) OR (machine learning) OR (deep learning) OR (neural network) | 113 | <https://zenodo.org/search?page=1&size=20&q=publication_date:%5B2020-01-01%20TO%202022-09-29%5D%20AND%20title:%20(oral%20disease)%20AND%20(artificial%20intelligence)%20OR%20(machine%20learning)%20OR%20(deep%20learning)%20OR%20(neural%20network)&sort=-publication_date> |
| zenodo | publication_date:[2020-01-01 TO 2022-09-29] AND title: (dental x-ray) AND (artificial intelligence) OR (machine learning) OR (deep learning) OR (neural network) | 48 | <https://zenodo.org/search?page=1&size=20&q=publication_date:%5B2020-01-01%20TO%202022-09-29%5D%20AND%20title:%20(dental%20x-ray)%20AND%20(artificial%20intelligence)%20OR%20(machine%20learning)%20OR%20(deep%20learning)%20OR%20(neural%20network)&sort=-publication_date> |
| zenodo |  | 122949 | <https://zenodo.org/search?q=teeth%20dataset%20panoramic&f=resource_type%3Adataset&f=access_status%3Aopen&l=list&p=1&s=10&sort=bestmatch> |
| Open Data Lab CN | CBCT | 7 | [https://opendatalab.org.cn/?keywords=%E9%94%A5%E5%BD%A2%E6%9D%9F&sort=all](https://opendatalab.org.cn/?keywords=锥形束&sort=all) |
| Open Data Lab CN | Periapical | 0 | [https://opendatalab.org.cn/?keywords=%E6%A0%B9%E5%B0%96%E7%89%87&sort=all](https://opendatalab.org.cn/?keywords=根尖片&sort=all) |
| Open Data Lab CN | Retroalveolar | 26 | [https://opendatalab.org.cn/?keywords=%E5%90%8E%E7%89%99%E6%A7%BD%E6%94%BE%E5%B0%84%E7%BA%BF%E7%89%87&sort=all](https://opendatalab.org.cn/?keywords=后牙槽放射线片&sort=all) |
| Open Data Lab CN | bitewing | 12 | [https://opendatalab.org.cn/?keywords=%E5%92%AC%E7%BF%BC%E7%89%87&sort=all](https://opendatalab.org.cn/?keywords=咬翼片&sort=all) |
| Open Data Lab CN | panoramic | 355 | [https://opendatalab.org.cn/?keywords=%E5%85%A8%E6%99%AF%E7%89%87%2C%20%E6%88%96%E8%80%85%20%E5%8F%A3%E8%85%94%E5%85%A8%E6%99%AFX%E7%BA%BF%E6%91%84%E5%BD%B1&sort=all](https://opendatalab.org.cn/?keywords=全景片%2C%20或者%20口腔全景X线摄影&sort=all) |
| Open Data Lab CN | caries | 0 | [https://opendatalab.org.cn/?keywords=%20%E9%BE%8B%E9%BD%BF&sort=all](https://opendatalab.org.cn/?keywords=%20龋齿&sort=all) |
| Open Data Lab CN | dentistry | 0 | [https://opendatalab.org.cn/?keywords=%E7%89%99%E7%A7%91&sort=all](https://opendatalab.org.cn/?keywords=牙科&sort=all) |

##

## Appendix Table 3. Codebook for the data extraction form

| Item | Form full text | Field Name | Data Type | Description |
| --- | --- | --- | --- | --- |
| Year of dataset publication |  | Year_Publication | Integer | Year the dataset was published |
| What are the main areas of the dataset/research? (Select all that apply) |  | Dataset_Focus | Multiple Choice | Main research areas of the dataset |
| Associated with a publication or paper? |  | Paper_Link | Yes/No | Is the dataset associated with a publication? |
| Country of origin (if not available, write NA) |  | Country_Origin | Text | Country dataset was collected in (or NA if unavailable) |
| DOI of the associated publication |  | Paper_DOI | Text | DOI of the associated publication (if applicable) |
| Data collection period |  | Start_Date | Date | Start date of dataset collection |
| Source of data acquisition (site) (multiple choices) |  | Acquisition_Source | Multiple Choice | Where images were acquired |
| Reason for image acquisition (multiple choices) |  | Acquisition_Reason | Multiple Choice | Reason images were acquired |
| Imaging modality (multiple choices) |  | Imaging_Modality | Multiple Choice | Type of imaging used |
| Please indicate whether the following dataset characteristics are reported | [Ethical approval for dataset publication] | Ethical_Approval | Yes/No/Not Sure | Was ethical approval stated for dataset publication? |
|  | [Participant consent] | Informed_Consent | Yes/No/Not Sure | Was informed consent from participants stated? |
|  | [Inclusion or exclusion criteria stated] | Inclusion_Criteria | Yes/No/Not Sure | Were inclusion/exclusion criteria reported? |
|  | [Segmentations] | Segmentations | Yes/No/Not Sure | Were image segmentations included? |
|  | [Lesion feature or image size annotations] | Lesion_Annotations | Yes/No/Not Sure | Were annotations for lesions/features included? |
|  | [Ground truth or gold standard method described] | Ground_Truth_Method | Text | How ground truth/gold standard was established |
|  | [Anonymisation strategy] | Anonymization_Strategy | Yes/No/Not Sure | Was an anonymization strategy described? |
|  | [Image acquisition device (e.g. Sirona, Germany)] | Acquisition_Device | Text/Not Sure | Image acquisition device used (if reported) |
|  | [License type of the dataset] | Dataset_License | Text/Not Sure | License type applied to the dataset |
|  | [Image processing] | Image_Processing_Adjustment | Yes/No/Not Sure | Were any image processing or adjustments reported? |
|  | [Gender ratio (males/females)] | Gender_Ratio | Text/Not Sure | Gender ratio of participants (males/females) |
|  | [Ethnicity] | Ethnicity_Reported | Yes/No/Not Sure | Was ethnicity reported? |
|  | Does the dataset include annotations? | Annotations_Included | Yes/No | Does the dataset include annotations? |
|  | Number of annotators | Num_Annotators | Integer (or 999) | Number of annotators |
|  | Type of Annotation (pixel-wise, box, label, etc) | Annotation_Type | Text | Type of annotation |
| About the annotators | [Is described the calibration or training of the annotators?] | Annotator_Calibration_Described | Yes/No | Was annotator training/calibration described? |
|  | [Is any metric related to the calibration of annotators reported (kappa, ICC, etc)?] | Annotator_Calibration_Metric | Yes/No | Was any calibration metric reported? |
|  | [Is the age of annotators reported?] | Annotator_Age | Text/Not Sure | Age of annotators (if reported) |
|  | [Is the experience or qualifications of the annotators described?] | Annotator_Experience | Text/Not Sure | Description of annotators' experience (if reported) |
|  | [Is the reporting of mechanisms/strategies to deal with disagreements included in the study?] | Disagreement_Strategies | Yes/No | Were strategies for dealing with disagreements reported? |
|  | [Is the software used for annotations described in the study?] | Annotation_Software | Text/Not Sure | Software used for annotations (if reported) |
| How was the ground truth / gold standard established in the study? |  |  | Text/Not Sure |  |
| Is the Number of patients in the dataset reported? |  |  | Yes/No |  |
| Number of patients in the dataset |  | Num_Patients | Integer | Number of patients in the dataset |
| Is the Number of images in the dataset reported? |  |  | Yes/No |  |
| Number of images in the dataset |  | Num_Images | Integer | Number of images in the dataset |
| Comments? (add any additional or relevant information) |  | Comments | Text | Any additional relevant information |

##

## Appendix table 4. Excluded hits. These included potential datasets but with no images or with less than 50 images, code repositories with no datasets, or papers with no data

| **Database** | **Dataset URL** | **Year** | **Area** | **Country** | **DOI associated** | **Patients** | **Images** |
| --- | --- | --- | --- | --- | --- | --- | --- |
| Pubmed | http://dx.doi.org/10.1038/s41415-022-4274-y;https://www.ncbi.nlm.nih.gov/pubmed/35562460;https://pubmed.ncbi.nlm.nih.gov/35562460 | 2022 | Caries | NA | 10.1016/j.oooo.2022.03.008 |  |  |
| Pubmed | http://dx.doi.org/10.1186/s40902-023-00382-w;https://www.ncbi.nlm.nih.gov/pubmed/36913002;https://www.ncbi.nlm.nih.gov/pmc/articles/PMC10011265;https://pubmed.ncbi.nlm.nih.gov/36913002 | 2023 | Oral surgery | NA | 10.1186/s40902-023-00382-w |  |  |
| Pubmed | https://www.mdpi.com/2075-4418/13/5/996;http://dx.doi.org/10.3390/diagnostics13050996;https://www.ncbi.nlm.nih.gov/pubmed/36900140;https://www.ncbi.nlm.nih.gov/pmc/articles/PMC10000385;https://pubmed.ncbi.nlm.nih.gov/36900140 | 2023 | Charting (teeth segmentation, etc) | NA | 10.3390/diagnostics13050996 | 888 | 888 |
| Pubmed | http://www.quintpub.com/journals/omi/abstract.php?iss2_id=1777&article_id=21816 | 2021 | Dental implants | NA |  |  |  |
| Pubmed | https://www.ajodo.org/article/S0889-5406(22)00656-4/fulltext | 2023 | Temporomandibular disorders | NA |  | 502 |  |
| Pubmed | http://dx.doi.org/10.1038/s41598-023-28442-1;https://www.ncbi.nlm.nih.gov/pubmed/36709380;https://www.ncbi.nlm.nih.gov/pmc/articles/PMC9884213;https://pubmed.ncbi.nlm.nih.gov/36709380 | 2023 | Other (Please specify) - prosthodontics | NA | 10.1038/s41598-023-28442-1 |  |  |
| Pubmed | http://dx.doi.org/10.1259/dmfr.20220225;https://www.ncbi.nlm.nih.gov/pubmed/36416666;https://www.ncbi.nlm.nih.gov/pmc/articles/PMC9793454;https://pubmed.ncbi.nlm.nih.gov/36416666 | 2023 | Oral pathology | NA | 10.1259/dmfr.20220225 |  |  |
| Pubmed | https://downloads.hindawi.com/journals/ecam/2022/3384209.pdf? | 2022 | Other (Please specify) - Tongue image | N/A | https://doi.org/10.1155/2022/3384209 | 8676 | 8676 |
| Pubmed | https://pdf.sciencedirectassets.com/271506/1-s2.0-S0957417418X00116/ | 2018 | Charting (teeth segmentation, etc) | NA | <https://doi.org/10.1016/j.compbiomed.2022.105829> |  |  |
| Pubmed | https://www.nature.com/articles/s41415-022-4274-y | 2022 | Caries | UK | [10.1038/s41415-022-4274-y](https://doi.org/10.1038/s41415-022-4274-y) |  |  |
| Pubmed | http://dx.doi.org/10.1111/scd.12832;https://www.ncbi.nlm.nih.gov/pubmed/36749021;https://pubmed.ncbi.nlm.nih.gov/36749021 | 2023 | Charting (teeth segmentation, etc) | USA | 10.1111/scd.12832 | 401958 |  |
| Pubmed | https://doi.org/10.3390/metabo13010037 | 2022 | Caries | israel | 10.3390/metabo13010037 | 66790 |  |
| Pubmed | https://www.ncbi.nlm.nih.gov/pmc/articles/PMC9530294/pdf/isd-52-275.pdf | 2022 | Other (Please specify) - Permanent tooth germ detection | Istanbul | 10.5624/isd.20220050 | 4518 | 50096 |
| Pubmed | http://dx.doi.org/10.2196/38640;https://www.ncbi.nlm.nih.gov/pubmed/36315222;https://www.ncbi.nlm.nih.gov/pmc/articles/PMC9664332;https://pubmed.ncbi.nlm.nih.gov/36315222 | 2022 | Caries | South Korea | 10.2196/38640 | 10000 | 10000 |
| Pubmed | http://dx.doi.org/10.1155/2023/6662911;https://www.ncbi.nlm.nih.gov/pubmed/36896411;https://www.ncbi.nlm.nih.gov/pmc/articles/PMC9991474;https://pubmed.ncbi.nlm.nih.gov/36896411 | 2023 | Oral pathology,Other (Please specify) - osteoporosis detection | Indonesia | 10.1186/s40902-023-00382-w | 102 |  |
| Pubmed | http://dx.doi.org/10.1007/s11424-022-2057-9;https://www.ncbi.nlm.nih.gov/pubmed/36258771;https://www.ncbi.nlm.nih.gov/pmc/articles/PMC9976655;https://pubmed.ncbi.nlm.nih.gov/36258771 | 2022 | Charting (teeth segmentation, etc) | China | 10.1007/s11424-022-2057-9 | 100 | 100 |
| Pubmed | http://dx.doi.org/10.3390/ijms24054557;https://www.ncbi.nlm.nih.gov/pubmed/36901988;https://www.ncbi.nlm.nih.gov/pmc/articles/PMC10003462;https://pubmed.ncbi.nlm.nih.gov/36901988 | 2023 | Oral surgery | Korea |  | 143 |  |
| Pubmed | https://pdf.sciencedirectassets.com/272201/1-s2.0-S1344622322X00060/1-s2.0-S1344622322001365/ | 2022 | Other (Please specify) - DENTAL AGE ASSESSMENT | Taiwan | 10.1016/j.legalmed.2022.102148 | 2052 | 2052 |
| Pubmed | http://dx.doi.org/10.1038/s41598-023-29890-5;https://www.ncbi.nlm.nih.gov/pubmed/36792647;https://www.ncbi.nlm.nih.gov/pmc/articles/PMC9931752;https://pubmed.ncbi.nlm.nih.gov/36792647 | 2023 | Oral pathology | South Korea | 10.1038/s41598-023-29890-5 | 216 |  |
| Pubmed | https://www.researchgate.net/profile/Min-Ji-Byon/publication/357432727_Evaluation_of_VGG-16_deep_learning_algorithm_for_dental_caries_classification/links/ | 2021 | Caries | South Korea | [10.11149/jkaoh.2021.45.4.227](http://dx.doi.org/10.11149/jkaoh.2021.45.4.227) |  |  |
| Pubmed | http://dx.doi.org/10.1038/s41598-022-21408-9;https://www.ncbi.nlm.nih.gov/pubmed/36209283;https://www.ncbi.nlm.nih.gov/pmc/articles/PMC9547920;https://www.nature.com/articles/s41598-022-21408-9;https://www.nature.com/articles/s41598-022-21408-9.pdf | 2022 | Oral surgery | Japan | 10.1038/s41598-022-21408-9 |  |  |
| Pubmed | https://www.mdpi.com/2076-3417/12/1/475;http://dx.doi.org/10.3390/app12010475 | 2022 | Oral surgery,Oral pathology | Korea | 10.3390/app12010475 |  |  |
| Google dataset search | https://ditto.ing.unimore.it/maxillo/ | 2022 | Dental implants,Oral surgery | Italy | 10.1109/ACCESS.2022.3144840 |  |  |
| Pubmed | https://www.ncbi.nlm.nih.gov/pubmed/34131266 | 2021 | Caries,Oral surgery | Netherlands |  | 253 | 253 |
| Pubmed | http://dx.doi.org/10.3122/jabfm.2022.220177R2;https://www.ncbi.nlm.nih.gov/pubmed/36948536;https://pubmed.ncbi.nlm.nih.gov/36948536 | 2023 | Caries | Canada | 10.3122/jabfm.2022.220177R2 | 26 |  |
| Pubmed | http://dx.doi.org/10.3290/j.ohpd.b2048359;https://www.ncbi.nlm.nih.gov/pubmed/34546013;https://pubmed.ncbi.nlm.nih.gov/34546013 | 2021 | Caries | Turkey | 10.3290/j.ohpd.b2048359 |  |  |
| Pubmed | http://dx.doi.org/10.3390/diagnostics11091572;https://www.ncbi.nlm.nih.gov/pubmed/34573914;https://www.ncbi.nlm.nih.gov/pmc/articles/PMC8469771 | 2021 | Oral surgery | Korea | 10.3390/diagnostics11091572 | 300 | 300 |
| Pubmed | http://dx.doi.org/10.1080/10255842.2023.2187671;https://www.ncbi.nlm.nih.gov/pubmed/36920276;https://pubmed.ncbi.nlm.nih.gov/36920276 | 2023 | Other (Please specify) - dental materials | India | 10.1080/10255842.2023.2187671 |  |  |
| Pubmed | http://dx.doi.org/10.3390/ijerph192215240;https://www.ncbi.nlm.nih.gov/pubmed/36429958;https://www.ncbi.nlm.nih.gov/pmc/articles/PMC9691188;https://pubmed.ncbi.nlm.nih.gov/36429958 | 2022 | Charting (teeth segmentation, etc) | Poland | 10.3390/ijerph192215240 | 300 | 300 |
| Pubmed | http://dx.doi.org/10.5624/isd.20220125;https://www.ncbi.nlm.nih.gov/pubmed/36605858;https://www.ncbi.nlm.nih.gov/pmc/articles/PMC9807788;https://pubmed.ncbi.nlm.nih.gov/36605858 | 2022 | Temporomandibular disorders | South Korea | 10.5624/isd.20220125 | 314 | 2634 |
| Pubmed | http://dx.doi.org/10.3390/diagnostics13050918;https://www.ncbi.nlm.nih.gov/pubmed/36900062;https://www.ncbi.nlm.nih.gov/pmc/articles/PMC10001077;https://pubmed.ncbi.nlm.nih.gov/36900062 | 2023 | Oral pathology | India | 10.3390/diagnostics13050918 |  |  |
| Pubmed | https://www.researchgate.net/publication/361081145_Automated_segmentation_of_head_CT_scans_for_computer-assisted_craniomaxillofacial_surgery_applying_a_hierarchical_patch-based_stack_of_convolutional_neural_networks | 2022 | Charting (teeth segmentation, etc) | Germany | 10.1007/s11548-022-02673-5 |  |  |
| Pubmed | http://dx.doi.org/10.3390/cancers15051421;https://www.ncbi.nlm.nih.gov/pubmed/36900210;https://www.ncbi.nlm.nih.gov/pmc/articles/PMC10001266;https://pubmed.ncbi.nlm.nih.gov/36900210 | 2023 | Oral pathology | USA and India | 10.3390/cancers15051421 |  |  |
| Pubmed | http://dx.doi.org/10.1038/s41598-022-20411-4;https://www.ncbi.nlm.nih.gov/pubmed/36253430;https://www.ncbi.nlm.nih.gov/pmc/articles/PMC9576767;https://pubmed.ncbi.nlm.nih.gov/36253430 | 2022 | Endodontics | China | 10.1038/s41598-022-20411-4 | 384 | 384 |
| Pubmed | http://dx.doi.org/10.3390/diagnostics12102537;https://www.ncbi.nlm.nih.gov/pubmed/36292226;https://www.ncbi.nlm.nih.gov/pmc/articles/PMC9600983;https://www.mdpi.com/journal/diagnostics | 2022 | Oral pathology | USA | 10.3390/diagnostics12102537 |  |  |
| Pubmed | http://dx.doi.org/10.1007/s11282-021-00538-2;https://www.ncbi.nlm.nih.gov/pubmed/34041639;https://www.ncbi.nlm.nih.gov/pmc/articles/PMC8741711;https://doi.org/10.1007/s11282-021-00538-2;https://link.springer.com/article/10.1007/s11282-021-00538-2 | 2022 | Charting (teeth segmentation, etc) | Japan | 10.1007/s11282-021-00538-2 |  |  |
| Pubmed | https://www.sciencedirect.com/science/article/abs/pii/S0141938221001219?via%3Dihub | 2022 | Oral pathology | China |  |  |  |
| Github | https://github.com/2006pmach/facial_attractiveness_prediction | 2006 | Other (Please specify) - Facial attractiveness | Switzerland | 10.1093/ejo/cjac016 | 40 | 960 |
| Pubmed | https://onlinelibrary.wiley.com/doi/10.1002/osi2.1177;http://dx.doi.org/10.1002/osi2.1177;https://onlinelibrary.wiley.com/doi/abs/10.1002/osi2.1177 | 2023 | Oral surgery | Japan | 10.1002/osi2.1177 | 400 | 400 |
| Pubmed | http://dx.doi.org/10.3389/fpubh.2022.1045815;https://www.ncbi.nlm.nih.gov/pubmed/36466455;https://www.ncbi.nlm.nih.gov/pmc/articles/PMC9713943;https://pubmed.ncbi.nlm.nih.gov/36466455 | 2022 | Charting (teeth segmentation, etc) | China | 10.3389/fpubh.2022.1045815 | 501 |  |
| Pubmed | http://dx.doi.org/10.1016/j.cmpb.2023.107465;https://www.ncbi.nlm.nih.gov/pubmed/36933315;https://pubmed.ncbi.nlm.nih.gov/36933315 | 2023 | Oral pathology | South Korea | 10.1016/j.cmpb.2023.107465 | 502 | 1709 |
| Pubmed | https://doi.org/10.3390/ijerph192215414 | 2022 | Dental implants,Other (Please specify) - cbct | saudi arabia |  |  |  |
| IEEE | https://ieeexplore.ieee.org/document/9630750 | 2021 | Endodontics | USA | 10.1109/EMBC46164.2021.9630750 |  |  |
| Pubmed | https://karger.com/cre/article/56/5-6/455/841972/Detection-of-Proximal-Caries-Lesions-on-Bitewing | 2022 | Caries | ChinA |  |  |  |
| Pubmed | https://bmcoralhealth.biomedcentral.com/articles/10.1186/s12903-022-02539-x | 2022 | Dental implants | KOrea |  |  |  |
| Pubmed | https://link.springer.com/article/10.1007/s11282-021-00584-w | 2022 | Other (Please specify) - Radiation | Japan |  |  |  |
| Pubmed | https://aap.onlinelibrary.wiley.com/doi/10.1002/JPER.21-0492 | 2022 | Dental implants | ChiNa |  |  |  |
| Pubmed | http://dx.doi.org/10.1177/00220345231152802;https://www.ncbi.nlm.nih.gov/pubmed/36919874;https://pubmed.ncbi.nlm.nih.gov/36919874 | 2023 | Caries | Thailand | 10.1177/00220345231152802 | 568 |  |
| Pubmed | https://www.sciencedirect.com/science/article/abs/pii/S1742706120306541?via%3Dihub | 2020 | Dental implants | Canada | 10.1109/EMBC46164.2021.9630750 |  |  |
| Pubmed | https://link.springer.com/article/10.1007/s10916-018-1051-1 | 2018 | Dental implants | Saudi Arabia | 10.1007/s10916-018-1051-1 |  |  |
| Pubmed | http://dx.doi.org/10.1038/s41598-021-81449-4;https://www.ncbi.nlm.nih.gov/pubmed/33479379;https://www.ncbi.nlm.nih.gov/pmc/articles/PMC7820274;https://pubmed.ncbi.nlm.nih.gov/33479379 | 2021 | Oral surgery | korea | 10.1038/s41598-021-81449-4 | 600 | 600 |
| Pubmed | https://www.mdpi.com/2075-4418/12/12/3081 | 2022 | Charting (teeth segmentation, etc) | Turkey | 10.3390/diagnostics12123081 |  |  |
| Pubmed | <https://academic.oup.com/dmfr/article/51/7/20220122/7261245?login=false> | 2022 | Periodontics | USA | 10.1259/dmfr.20220122 |  |  |
| Pubmed | http://dx.doi.org/10.3390/s22020637;https://www.ncbi.nlm.nih.gov/pubmed/35062599;https://www.ncbi.nlm.nih.gov/pmc/articles/PMC8777593;https://www.mdpi.com/journal/sensors | 2022 | Charting (teeth segmentation, etc) | Poland | 10.3390/s22020637 | 619 | 619 |
| Pubmed | https://www.sciencedirect.com/science/article/abs/pii/S0021929008006295?via%3Dihub | 2009 | Dental implants | Singapore | 10.1016/j.jbiomech.2008.12.001 |  |  |
| Pubmed | https://doi.org/10.1007/s11042-023-14746-x;http://dx.doi.org/10.1007/s11042-023-14746-x;https://link.springer.com/article/10.1007/s11042-023-14746-x | 2023 | Charting (teeth segmentation, etc),Other (Please specify) - Forensics | Turkey | 10.1007/s11042-023-14746-x | 64 | 640 |
| Pubmed | <https://www.ncbi.nlm.nih.gov/pmc/articles/PMC9516607/> | 2022 | Oral surgery | Netherlands | 10.1177/00220345221117745 | 7 |  |
| Pubmed | https://link.springer.com/article/10.1007/s11042-023-14435-9 | 2023 | Charting (teeth segmentation, etc) | brazil |  |  |  |
| Pubmed | http://dx.doi.org/10.1186/s12903-022-02614-3;https://www.ncbi.nlm.nih.gov/pubmed/36476146;https://www.ncbi.nlm.nih.gov/pmc/articles/PMC9730580;https://pubmed.ncbi.nlm.nih.gov/36476146 | 2022 | Oral surgery | South Korea | 10.1186/s12903-022-02614-3 | 724 |  |
| Pubmed | https://www.mdpi.com/2075-4418/13/2/202 | 2023 | Caries | Turkey |  |  |  |
| Pubmed | https://doi.org/10.4103/1735-3327.369629 | 2023 | Oral surgery | Iran |  |  |  |
| Pubmed | https://link.springer.com/article/10.1007/s11042-023-14746-x | 2023 | Charting (teeth segmentation, etc) | turkey |  |  |  |
| Pubmed | http://dx.doi.org/10.1177/09544119231162682;https://www.ncbi.nlm.nih.gov/pubmed/36939160;https://pubmed.ncbi.nlm.nih.gov/36939160 | 2023 | Periodontics | Turkey | 10.1177/09544119231162682 | 8 | 87 |
| Pubmed | http://dx.doi.org/10.2196/32921;https://www.ncbi.nlm.nih.gov/pubmed/34529582;https://www.ncbi.nlm.nih.gov/pmc/articles/PMC8571694;https://pubmed.ncbi.nlm.nih.gov/34529582 | 2021 | Caries | United States of America | 10.2196/32921 | 84 |  |
| Pubmed | http://dx.doi.org/10.1016/j.cmpb.2023.107467;https://www.ncbi.nlm.nih.gov/pubmed/36921464;https://pubmed.ncbi.nlm.nih.gov/36921464 | 2023 | Temporomandibular disorders | South Korea |  |  |  |
| Pubmed | http://dx.doi.org/10.1007/s11282-023-00677-8;https://www.ncbi.nlm.nih.gov/pubmed/36920598;https://www.ncbi.nlm.nih.gov/pmc/articles/6592580;https://pubmed.ncbi.nlm.nih.gov/36920598 | 2023 | Charting (teeth segmentation, etc) | Turkey | 10.1007/s11282-023-00677-8 |  |  |
| Pubmed | http://dx.doi.org/10.1111/joor.13440;https://www.ncbi.nlm.nih.gov/pubmed/36843391;https://pubmed.ncbi.nlm.nih.gov/36843391 | 2023 | Temporomandibular disorders | Austrailia |  |  |  |
| Pubmed | http://dx.doi.org/10.1093/pnasnexus/pgac239;https://www.ncbi.nlm.nih.gov/pubmed/36712365;https://www.ncbi.nlm.nih.gov/pmc/articles/PMC9802336;https://pubmed.ncbi.nlm.nih.gov/36712365 | 2022 | Caries | Austrailia | 10.1093/pnasnexus/pgac239 | 88 |  |
| Pubmed | http://dx.doi.org/10.1038/s41598-023-27950-4;https://www.ncbi.nlm.nih.gov/pubmed/36639691;https://www.ncbi.nlm.nih.gov/pmc/articles/PMC9839705;https://pubmed.ncbi.nlm.nih.gov/36639691 | 2023 | Charting (teeth segmentation, etc) | South Korea | 10.1038/s41598-023-27950-4 | 910 | 910 |
| Pubmed | https://pubmed.ncbi.nlm.nih.gov/36758590/ | 2022 | Endodontics | China |  |  |  |
| Pubmed | http://dx.doi.org/10.1186/s12903-023-02817-2;https://www.ncbi.nlm.nih.gov/pubmed/36803132;https://www.ncbi.nlm.nih.gov/pmc/articles/PMC9938587;https://pubmed.ncbi.nlm.nih.gov/36803132 | 2023 | Charting (teeth segmentation, etc) | China | https://survey.zohopublic.com/zs/ZuBUg8 | 9586 |  |
| Pubmed | http://dx.doi.org/10.1111/clr.14063;https://www.ncbi.nlm.nih.gov/pubmed/36906917;https://onlinelibrary.wiley.com/doi/10.1111/clr.14063;https://onlinelibrary.wiley.com/doi/abs/10.1111/clr.14063 | 2023 | Charting (teeth segmentation, etc) | Belgium | 10.1111/clr.14063 |  |  |
| Pubmed | http://dx.doi.org/10.1177/00220345231152802;https://www.ncbi.nlm.nih.gov/pubmed/36919874;https://pubmed.ncbi.nlm.nih.gov/36919874 | 2023 | Oral surgery | China | 10.1177/00220345231152802 |  |  |
| Pubmed | http://dx.doi.org/10.3390/diagnostics13010110;https://www.ncbi.nlm.nih.gov/pubmed/36611402;https://www.ncbi.nlm.nih.gov/pmc/articles/PMC9818323;https://pubmed.ncbi.nlm.nih.gov/36611402 | 2023 | Oral surgery,Oral pathology | China | 10.3390/diagnostics13010110 |  |  |
| Pubmed | http://dx.doi.org/10.1080/00016357.2022.2158929;https://www.ncbi.nlm.nih.gov/pubmed/36548872;https://pubmed.ncbi.nlm.nih.gov/36548872 | 2022 | Endodontics | Denmark | 10.1080/00016357.2022.2158929 |  |  |
| Pubmed | http://dx.doi.org/10.3389/fgene.2022.1041524;https://www.ncbi.nlm.nih.gov/pubmed/36457739;https://www.ncbi.nlm.nih.gov/pmc/articles/PMC9705329;https://pubmed.ncbi.nlm.nih.gov/36457739 | 2022 | Periodontics | China | 10.3389/fgene.2022.1041524 |  |  |
| Pubmed | http://dx.doi.org/10.1109/TMI.2022.3222388;https://www.ncbi.nlm.nih.gov/pubmed/36378797;https://pubmed.ncbi.nlm.nih.gov/36378797 | 2022 | Charting (teeth segmentation, etc) | China | 10.1109/TMI.2022.3222388 |  |  |
| Pubmed | http://dx.doi.org/10.3390/ma15207246;https://www.ncbi.nlm.nih.gov/pubmed/36295314;https://www.ncbi.nlm.nih.gov/pmc/articles/PMC9610509;https://pubmed.ncbi.nlm.nih.gov/36295314 | 2022 | Other (Please specify) - Prosthodontics | Russia | 10.3390/ma15207246 |  |  |
| Pubmed | http://dx.doi.org/10.3390/healthcare10102089;https://www.ncbi.nlm.nih.gov/pubmed/36292536;https://www.ncbi.nlm.nih.gov/pmc/articles/PMC9601705;https://pubmed.ncbi.nlm.nih.gov/36292536 | 2022 | Charting (teeth segmentation, etc) | China | 10.3390/healthcare10102089 |  |  |
| Mendeley | https://data.mendeley.com/datasets/hxt48yk462/2 | 2022 | Other (Please specify) - systemic disease classification (Osteoarthritis) | Iran | 10.22266/ijies2022.1231.19 | 116 | 6000 |
| Pubmed | https://doi.org/10.1186/s12903-020-1062-4 | 2020 | Other (Please specify) - Crowns | germany | 10.1186/s12903-020-1062-4 | 90 |  |
| Pubmed | https://onlinelibrary.wiley.com/doi/10.1111/jcpe.13797 | 2023 | Oral surgery | italy | [10.1111/jcpe.13797](https://doi.org/10.1111/jcpe.13797) | 49 |  |
| Pubmed | https://bmcoralhealth.biomedcentral.com/articles/10.1186/s12903-023-02844-z | 2023 | Other (Please specify) - Orthognathic Surgery | China | 10.1186/s12903-023-02844-z | 383 |  |
| Pubmed | https://link.springer.com/article/10.1007/s10439-022-03056-2 | 2022 | Temporomandibular disorders | Taiwan |  | 32 | 50 |
| Pubmed | https://www.mdpi.com/2306-5354/10/2/245 | 2023 | Caries | korea |  | 22288 |  |
| Pubmed | https://www.nature.com/articles/s41598-021-81449-4 | 2021 | Oral surgery | south korea |  | 1053 | 600 |
| Pubmed | https://isdent.org/DOIx.php?id=10.5624/isd.20220105 | 2022 | Periodontics | Indonesia | 10.5624/isd.20220105 | 100 | 1000 |
| Pubmed | http://dx.doi.org/10.1155/2022/9333406;https://www.ncbi.nlm.nih.gov/pubmed/36245930;https://www.ncbi.nlm.nih.gov/pmc/articles/PMC9553657;https://pubmed.ncbi.nlm.nih.gov/36245930 | 2022 | Oral pathology,Endodontics | China |  |  |  |
| Pubmed | http://dx.doi.org/10.1038/s41598-022-20411-4;https://www.ncbi.nlm.nih.gov/pubmed/36253430;https://www.ncbi.nlm.nih.gov/pmc/articles/PMC9576767;https://pubmed.ncbi.nlm.nih.gov/36253430 | 2022 | Endodontics | China | 10.1038/s41598-022-20411-4 |  |  |
| Pubmed | http://dx.doi.org/10.3390/healthcare10102089;https://www.ncbi.nlm.nih.gov/pubmed/36292536;https://www.ncbi.nlm.nih.gov/pmc/articles/PMC9601705;https://pubmed.ncbi.nlm.nih.gov/36292536 | 2022 | Charting (teeth segmentation, etc) | China | 10.3390/healthcare10102089 |  |  |
| Github | <https://github.com/patmo141/d3tool_tooth_segmentation> |  |  |  |  |  |  |
| Github | <https://github.com/patmo141/d3tool_tooth_segmentation> |  |  |  |  |  |  |
| Github | <https://github.com/Shrey09/Tooth_Detection/tree/master/ToothDetection> |  |  |  |  |  |  |
| Pubmed | <https://www.sciencedirect.com/science/article/pii/S0169260722004771> | 2022 | Other (Please specify) - Tongue | No | [10.1016/j.cmpb.2022.107096](https://doi.org/10.1016/j.cmpb.2022.107096) | 42 | 42 |
| ArXiv | http://arxiv.org/abs/2303.06500 | 2023 | Oral surgery | China | 10.48550/arXiv.2303.06500 |  |  |
| Pubmed | https://www.frontiersin.org/articles/10.3389/fninf.2022.1067040/full | 2022 | Dental implants | Russia | 10.3389/fninf.2022.1067040 | 1646 |  |
| Pubmed | https://doi.org/10.1016/j.phro.2019.05.001 | 2019 | Other (Please specify) - Radiography | canada | 10.1016/j.phro.2019.05.001 | 131 |  |
| Github | <https://github.com/qinxin99/qinxini> | 2023 | Dental panoramic radiography, impacted teeth, periodontitis, dental caries | China | 10.1007/s10278-024-00972-8 | No | 6580 |
| Github | <https://github.com/IvisionLab/OdontoAI-Open-Panoramic-Radiographs> | 2023 | Focus on dental panoramic radiographs with applications in instance segmentation and human-in-the-loop labeling. | Brazil | 10.1080/21681163.2022.2157747 |  | 4000 |
| Pubmed | 10.1007/s11282-019-00409-x | 2020 |  |  | 10.1007/s11282-019-00409-x |  |  |
| Pubmed | [10.1109/TIPTEKNO.2017.8238075](https://doi.org/10.1109/TIPTEKNO.2017.8238075) | 2017 |  |  | [10.1109/TIPTEKNO.2017.8238075](https://doi.org/10.1109/TIPTEKNO.2017.8238075) |  |  |
| Pubmed | 10.1259/dmfr.20180051 | 2019 |  |  | 10.1259/dmfr.20180051 |  |  |
| Pubmed | 10.1109/SIBGRAPI.2018.00058 | 2018 |  |  | 10.1109/SIBGRAPI.2018.00058 |  |  |
| Pubmed | [10.1007/s11282-019-00418-w](https://doi.org/10.1007/s11282-019-00418-w) | 2021 |  |  | [10.1007/s11282-019-00418-w](https://doi.org/10.1007/s11282-019-00418-w) |  |  |
| Pubmed | [10.1016/j.media.2016.02.004](https://doi.org/10.1016/j.media.2016.02.004) | 2016 |  |  | [10.1016/j.media.2016.02.004](https://doi.org/10.1016/j.media.2016.02.004) |  |  |
| kaggle | <https://www.kaggle.com/datasets/pushkar34/teeth-dataset> |  |  |  |  |  |  |
| Zenodo | [https://zenodo.org/records/7812323#.ZDQE1uxBwUG](https://zenodo.org/records/7812323#.ZDQE1uxBwUG) | 2023 | Yes | Italy |  |  |  |
| Grand-Challenge | <https://ditto.ing.unimore.it/toothfairy/> | 2023 | Oral Surgery | Italy |  | 347 | 347 |
